# On Apoplexia Hydrocephalica, in Answer to Mr. Weaver

**Published:** 1806-08

**Authors:** 


### *To the Editors of the Medical ana Physical Journal.*

Gentlemen,

Every liberal mind must feel greatly indebted to those intelligent practitioners, who, through the medium of your valuable Journal, present to public notice any novel and interesting discovery in the healing art; actuated, as they must be, by a laudable zeal for the alleviation of human misery, and anxious to contribute their mite to that philanthropic end. Such, I am well aware, is the intention of your correspondent, Mr. Weaver, in his case of apoplexia hydrocephalica (No. 86). I cannot, however, conform to his principles; and as temperate discussion is the nurse of improvement, I trust the following remarks will be attributed to a similar motive.

To the observations preliminary to the case, I cannot but assent; not so, however, to the treatment of it; nor, can I agree with him in the position, "that if hydrocephalus is in general undertaken previous to the second stage, it will not, in a majority of instances, resist the usual mode of treatment;" although, I think, by due attention and acumen in the practitioner, in ascertaining the disease at his first attendance, and by active means *then* zealously employed, it may very often be arrested in its progress; but, if that is to be the case, if its gigantic strides are to be impeded, it must be accomplished by different and more active means than those employed by Mr. W.

At his first visit, when there appeared convulsions, was there no flushed face, no contracted pupil, and fever? And was not the nausea and vomiting indicative of the irritability of the common sensorium? If so, why an emetic; which, by propelling more blood to the head, by adding fuel to the fire, must evidently have increased the disease:—bleeding, general and topical, the cold affusion to the head, brisk saline purges, and an active antiphlogistic plan, I should have thought more likely to have resolved the disease, if the resolution of it was possible.

On the second visit, the languid and inactive state of the child, the heavy and burthensome appearance of the head, the dilated pupils, and the frequent startings and screamings, certainly indicated pressure upon the brain, and that the secondary disease was formed; and then the case was one of those fortunate few, where the absorbent powers, being either the effort of nature, or roused by art, are equivalent to a removal of the exciting cause.

Mr. W. however, observes, "that by a happy combination of two powerful agents, digitalis and calomel, much good was done; and which, he is persuaded, neither of them, *per se*, could have effected.

I must confess myself surprised at reading, that on the fifth day of the exhibition of the digitalis (the tenth of the the disease) eight grains were given to a child, three years old, and combined with 16 grains of calomel;—but much more so was I at finding, that the next day (the 11th), the child was improving both in *health and strength!* As I could not reconcile the above with my own experience in the sedative and debilitating effects of that powerful drug; and which I have in no instance whatever been able to exhibit, to any thing like the extent mentioned by Mr. W. I think it very desirable to ascertain, whether it was genuine and possessed of its wonted powers; as, if it was not so, but inert, the precedent, from a similar quantity being given in a similar case, may prove unfortunate indeed!—Should you favour this letter with an insertion, it will, I trust, meet Mr. W's eye; who, I am very sure, will explain the point, if he finds any explanation necessary.

I am, &c.

